# Analysis of *TET2* mutations in paroxysmal nocturnal hemoglobinuria (PNH)

**DOI:** 10.1186/s40164-019-0142-0

**Published:** 2019-08-21

**Authors:** Camille Lobry, Ashish Bains, Leah B. Zamechek, Sherif Ibrahim, Iannis Aifantis, David J. Araten

**Affiliations:** 1 Institut National de la Santé et de la Recherche Medicale (INSERM) U1170, Institut Gustave Roussy, 94805 Villejuif, France; 2 Pathology and Laboratory Medicine, Temple University, 3401 North Broad Street, Philadelphia, PA 19140 USA; 3 Columbia University Medical Center, 1130 St. Nicholas Avenue, Room 901, New York City, USA; 4 Cairo Diagnostics, 244 Westchester Avenue, West Harrison, NY 10604 USA; 5 Department of Pathology, NYU School of Medicine, 550 First Avenue, New York, NY 10016 USA; 6 Division of Hematology, Laura and Isaac Perlmutter Cancer Center, NYU Langone Health and the NYU School of Medicine, 240 East 38th Street, 19th Floor, New York, NY 10016 USA; 7 Division of Hematology, New York VA Medical Center, 423 East 23rd Street, New York, NY 10010 USA

**Keywords:** Paroxysmal nocturnal hemoglobinuria (PNH), TET2 gene, Myeloproliferative disorders, Aplastic anemia, Somatic mutations

## Abstract

**Background:**

Large clonal populations of cells bearing *PIG*-*A* mutations are the sine qua non of PNH, but the *PIG*-*A* mutation itself is insufficient for clonal expansion. The association between PNH and aplastic anemia supports the immune escape model, but not all PNH patients demonstrate a history of aplasia; therefore, second genetic hits driving clonal expansion have been postulated. Based on the previous identification of *JAK2* mutations in patients with a myeloproliferative/PNH overlap syndrome, we considered *TET2* as a candidate gene in which mutations might be contributing to clonal expansion.

**Methods:**

Here we sequenced the *TET2* and *JAK2* genes in 19 patients with large PNH clones.

**Results:**

We found one patient with a novel somatic nonsense mutation in *TET2* in multiple hematopoietic lineages, which was detectable upon repeat testing. This patient has had severe thromboses and has relatively higher peripheral blood counts compared with the other patients—but does not have other features of a myeloproliferative neoplasm.

**Conclusions:**

We conclude that mutations in *TET2* may contribute to clonal expansion in exceptional cases of PNH.

## Background

Paroxysmal nocturnal hemoglobinuria (PNH) is characterized by complement mediated hemolysis, immune mediated marrow failure, and an expansion in the marrow of a stem cell with an acquired somatic mutation in *PIG*-*A* [1]. This gene is essential for the biosynthesis of glycosylphosphatidylinositol (GPI), and circulating cells derived from the PNH clone are missing all GPI-linked proteins, including the complement inhibitors CD55 and CD59 [2]. The lack of these proteins sensitizes the red cell to complement mediated lysis. Platelets derived from the mutant stem cell clone have the same surface defect as red cells, but here the effect of uninhibited complement may primarily lead to a state of activation, explaining the marked hypercoagulable state seen in this disorder [3–6]. Other contributing factors may involve a decrease in fibrinolysis due to abnormal post-translational modification of the GPI-linked uPAR receptor [7], increased thrombin generation on platelet-derived microparticles [8–10], and defects related to the GPI-linked tissue factor pathway inhibitor [11].

Normal individuals harbor occult circulating blood cells with *PIG*-*A* mutations [12], and in mice, *Pig*-*A* disruption is not sufficient to drive clonal expansion [13], suggesting that clonal expansion depends on additional mechanisms. The immune escape model [14] posits that the *PIG*-*A* gene mutation represents the “first hit”, and aplastic anemia (AA)—which selects for GPI (−) stem cells—represents the necessary “second hit”. In support of this model, the GPI-anchor can fit into the groove of the HLA-like molecule CD1d [15], there is recent evidence that GPI itself may be the auto-antigen [16], and lymphocyte cultures can be raised to selectively kill GPI (+) cells [17]. Furthermore, the immune escape model is supported by the demonstration of oligoclonal T cell expansions [18] and an HLA DR-15/16 [19] association. Although not all patients with PNH develop AA, many have a subclinical form of stem cell loss [20].

There are, however, features of PNH that cannot be so readily explained by the immune escape model, such as the observation that rare patients will develop acute leukemia [21], advanced myelodysplasia [22], or features of a myeloproliferative neoplasm (MPN) [23]. Therefore, there is long-standing interest in identifying second *genetic* hits. In 24% of patients with PNH, we have found an abnormal karyotype [22], but this frequently regresses, despite persistence of the PNH clone. Two exceptional patients have been reported with an abnormality involving chromosome 12 [24], leading to over-expression of *HMGA2* (on chromosome 12q14.3), which results in a myeloproliferative syndrome when overexpressed in mice [25]. We have reported that activating *JAK2*^V617F^ mutations represent a second genetic hit seen occasionally in PNH [26], and this phenomenon probably accounts for the case reports from the 1970’s of patients with a positive HAM test and an MPN [23].

We have now hypothesized that, as for *JAK2*, mutations in *TET2* could also represent a second genetic hit. *TET2* encodes an enzyme of 2002 amino acids that is involved in the conversion of methylcytosine to hydroxymethylcytosine, using α-ketoglutarate as a co-factor [27–29]. This is likely to induce changes in gene expression patterns as a consequence of altered cytosine methylation, leading to a proliferation of myeloid cells: indeed, homozygous or heterozygous *Tet2* inactivation in mice results in an advantage for stem cells in competitive reconstitution experiments [30, 31]. Mono-allelic mutations in *TET2* have been found in about 12% of MPNs, about 20% of cases of MDS, as well as in CMMoL, AML, and mastocytosis [32]. Because *TET2* functions as a haploinsufficient tumor suppressor gene, it has an unusual combination of features: a broad spectrum of somatic inactivating mutations are pathogenic, and only one allele needs to be mutated. Indeed, *PIG*-*A*, because it is X-linked, represents the other most prominent example of a gene with both of these features. We therefore investigated whether *TET2* mutations would be found as a second genetic hit in patients with PNH.

## Methods

Patients diagnosed with PNH were recruited to an IRB-approved protocol after providing written informed consent. Diagnostic flow cytometry for PNH was performed by staining whole blood with FITC conjugated anti-CD59 for red cells, PE-conjugated anti-CD24 and Alexa-488 conjugated to FLAER for granulocytes and identification by FSC/SSC (on a log–log scale for red cells). For DNA extraction, granulocytes were isolated from whole blood by sedimentation in 6% hetastarch, centrifugation over ficoll, followed by osmotic lysis of red cells. We have included in this analysis only patients with more than 75% PNH granulocytes.

Extracted DNA from granulocytes was subjected to whole genome amplification, followed by bi-directional sequencing using a dye terminator approach using previously published primers [33]. To isolate separate GPI (+) and GPI (−) lymphocyte populations as well as monocyte populations, buffy coat cells were incubated with FLAER-Alexa 488 (which directly binds to GPI) and anti-CD33-PE, followed by sorting on a DakoCyomation MoFlo instrument. To isolate nucleated red cells, cells from the buffy coat layer were sorted based on their expression of glycophorin A and based on their FSC/SSC properties using the red cell settings.

## Results

Upon sequencing of the *TET2* gene in our cohort of patients, we identified the presence of several previously reported SNPs. In 11 out of 19 patients, we identified the c.5284A>G; p.I1762V variant, with an allele frequency of 34% compared with 22% in the NCBI dbSNP database. In 6 of the patients, we identified the c.5162T>G; p.L1721W variant, with an allele frequency of 15.8%, compared with 9.2% in the database. In 3 patients, we identified the c.1088C>T; p.P363L variant, with an allele frequency of 7.9%, compared with 3% in the database. None of these differences were statistically significant. One remarkable patient (patient 14), however, was heterozygous for all three of these SNPs– and was also heterozygous for a nonsense mutation, c.2697T>A; pY899X, which has not been previously reported (Table 1).

**Table 1:** Summary of clinical and genetic results in a cohort of 19 patients with PNH

Pt	Age/Gender	WBC (× 10^−3^/μl)	HGB (gm/dl)	PLT (x 10^−3^/μl)	ANC (x 10^−3^/μl)	Abs.Retic (per μl)	% PNH RBC (III)	% PNH RBC (PNH II)	%PNH PMN’s	Thrombosis	History of AA	Eculizumab	Mutation 1	Mutation 2	Mutation 3	Mutation 4
1	65F	4.5	9.6	165	2.5	299,000	82	1.6	99	No	No	Yes				
2	46M	3.4	10.6	35	2.2	60,800	13	2	92	Yes	Yes	Yes	I1762V	L1721W		
3	50F		11.7	59	5.8		18	52	92	Yes	No	Yes				
4	32M	3.7	10.5	157	2.5	88,000	26	3.8	97	Yes	No	Yes	I1762V			
5	37F	3	10.8	203	0.9	208,000	94		100	No	No	Yes				
6	54M	3.7	10.9	122	2.2	132,000	31		78	No	No	No	P363L	L1721W		
7	48M	3.7	9.6	103	2.2	91,000	32	2	89	No	Yes	Yes	G355D	I1762V	I1762V	
8	25F	2.6	8.5	74	1.3	114,000	22	2	91	No	No	Yes	I1762V	I1762V		
9	47M	5.1	9.4	175	3.1	117,000	21	4	95	No	Yes	Yes	I1762V			
10	48F	3	9.1	152	1.6	206,000	77		100	No	Yes	Yes				
11	52M	9.5	11.5	136	8.6	180,000	24		83	No	No	No	I1762V			
12	39F	5.1	9	361	1.7	247,000	91	1.5	97	No	Yes	Yes	I1762V			
13	28M	4.4	11.1	132	2.1	195,000	58		88	Yes	Yes	Yes	G355D	I1762V		
14	52F	7.7	9.9	321	3.5	247,000	99	1	> 99	Yes	No	Yes	P363L	*Y899X*	L1721W	I1762V
15	21F	3.6	12.1	132	1.6	126,000	51	5	80	No	No	No	L1721W			
16	27F	2.4	7.9	123	1.5	46,000	18	20	98	Yes	Yes	Yes	Y867H	P1723S	I1762V	H1778R
17	20F	3.7	8.2	96	1.7	317,000	48		99	No	No	Yes				
18	61F	4.9	9.2	239	2.2	114,000	44	12	88	No	Yes	Yes	P363L	L1721W		
19	55M	2.8	10.8	95	1.8	198,000	85	3	98	Yes	Yes	Yes	L1721W	I1762V		

The presence of common polymorphisms in *TET2* is indicated in plain text, and the novel nonsense mutation is indicated in italics. Two patients were homozygous for the I1762V polymorphism. The *JAK2*^V617F^ mutation was not identified in any of these patients; analysis of *JAK2* exon 12 was performed for patients 1–14 and patients 16–19, and no mutations were found

**Fig. 1:**
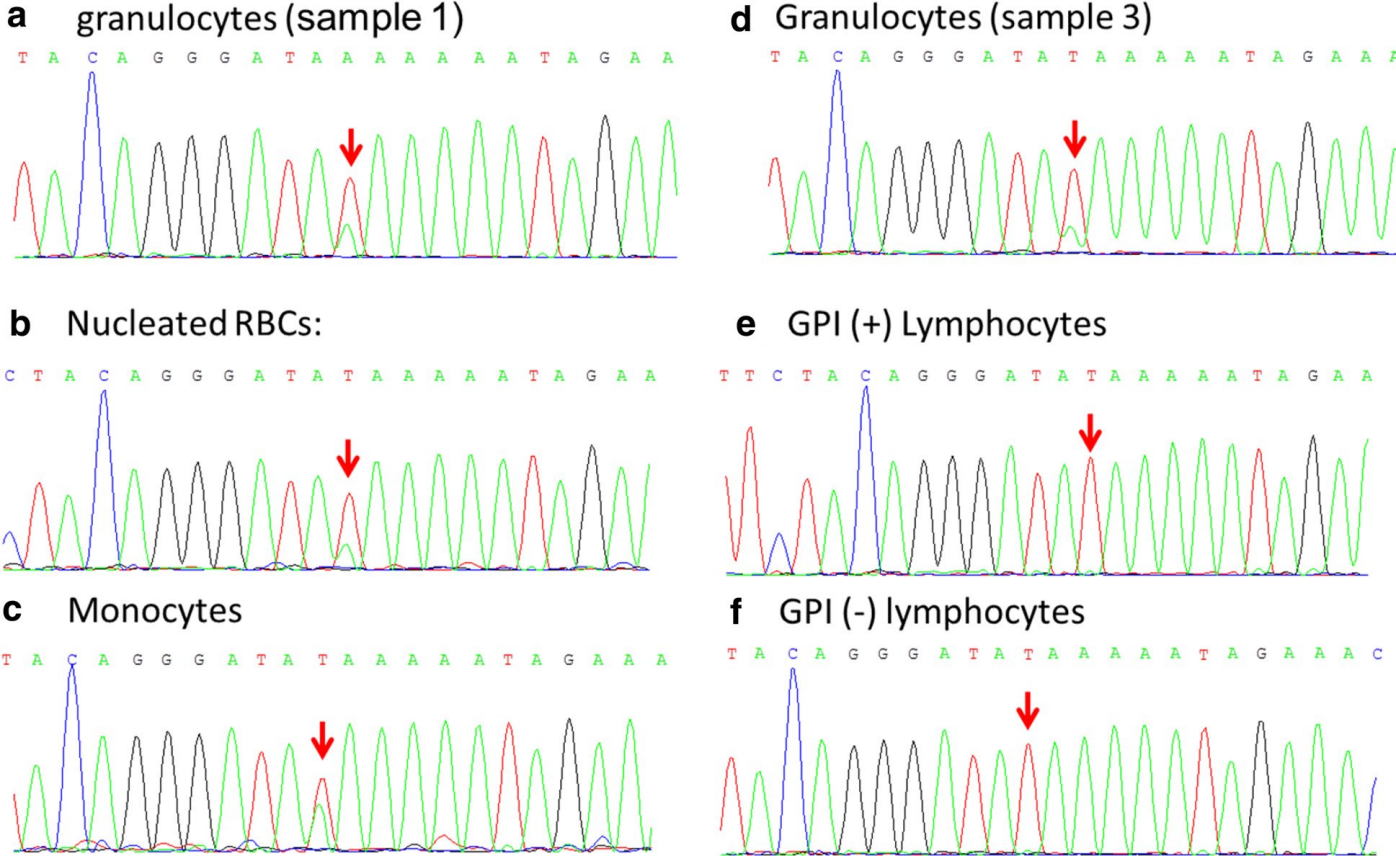
Electropherograms demonstrate the presence of the 2697T>A (Y899X) mutation in blood cells from patient 14. **a** Sequences amplified from granulocytes from the initial blood sample. **b**–**f** Sequences amplified from cells from the third sample obtained 16 months after the initial blood sample: **b** sorted glycophorin A-positive nucleated red cells; **c** sorted CD33-positive Monocytes; **d** Granulocytes; **e** sorted FLAER-positive lymphocytes; **f** sorted FLAER-negative lymphocytes. The arrow indicates the peak at the 2697 position. The highest proportion of mutant sequences was seen amongst the monocytes

A set of repeat sequencing reactions confirmed the presence of the 2697T>A mutation in granulocyte samples taken 8 months and then again 16 months after the initial sample (Fig. 1). The mutation was not found in either the sorted GPI (+) or GPI (−) lymphocytes, essentially ruling out a germline mutation. The *TET2* mutation was found in nearly a 1:1 ratio amongst the sorted monocytes, and was found in a lower ratio amongst sorted nucleated red cells and granulocytes.

In this patient, almost 100% of the granulocytes and red cells display the PNH phenotype. Using a method that we have recently developed (manuscript in preparation)—we have determined that nearly 100% of her platelets are GPI-negative, as well as approximately 30% of the lymphocytes. There was a history of severe thrombotic complications (involving the middle hepatic vein, portal vein, extremity DVT and PE, and a spontaneous hemorrhagic CVA that may have been secondary to a thrombotic event). This patient has a persistently high reticulocyte count and is unusual in that there were elevated blood counts compared with the other patients in the cohort (WBC of 7.7 vs median of 3.7, platelet count of 321 vs median of 131). Of the WBC’s, 4% were nucleated red cells. Interestingly, this patient’s absolute monocyte count ranged from 2 to 4 times above the upper limit of normal. PNH was first confirmed by flow cytometry in 1999, in the setting of hemolysis and a history of having had thrombosis. This patient had been transfusion dependent with an LDH 9 times the upper limit of normal prior to starting eculizumab in 2008. This patient has not had new thromboses in at least the past 18 years while on warfarin. Based on the timeline of symptoms, it is likely that this patient has had PNH since the 1980’s, without having ever had aplastic anemia. While there are no other features of a myeloproliferative neoplasm, the natural history of this patient’s presentation may have been altered due to a splenectomy for the presumptive diagnosis of ITP prior to the diagnosis of PNH.

## Discussion

In a series of 19 patients with “classic” PNH, we have found a *TET2* mutation only in patient 14, and in contrast to a separate series of patients [26], activating *JAK2* mutations were not found in any patient here. This confirms that the PNH/MPN overlap syndrome is a rare phenomenon, and we conclude that *TET2* mutations are not a common feature of PNH. Patient 14 had an unusual occurrence of 4 different base pair substitutions; while 3 of these are known polymorphisms, the Y899X substitution is very likely to be affecting the enzymatic activity of the TET2 protein, given that it is a truncating mutation, occurring at the 3′ end of exon 3, in a region where chain terminating mutations have been previously reported in patients with myeloid disorders [34].

The fact that the same mutation was seen in granulocytes, monocytes, and nucleated red cells, and was still present 16 months later suggests that the mutation resides in a long-lived stem cell clone with a potential for multilineage hematopoiesis. Although the patient does not have a clinically evident MPN, it is interesting that the WBC is higher than all of the other patients in the cohort, and this patient’s platelet count was the second highest of the 19 patients. Interestingly, this patient’s absolute monocyte count ranged from 2 to 4 times above the upper limit of normal, which is not a typical finding in PNH. The relative elevation in the blood counts and a particular increase in monocytes, however, is very much consistent with findings from mice that are genetically haploinsufficient for *Tet2* [31].

In mice, haploinsufficiency for *Tet2* also produces an advantage for stem cells based on in vivo competitive repopulation studies [31]. Therefore, we posit that the *TET2* mutation identified here conferred a survival advantage, perhaps in the context of an injured marrow. Conversely, given that 5.6% of healthy women with clonal hematopoiesis over the age of 65 have monoallelic inactivating *TET2* mutations [35], one might question whether this patient’s *TET2* mutation could be simply a part of that process. However, among 96 healthy women under the age of 60 who were known to have clonal hematopoiesis, *TET2* mutations were not found [35], and of note, patient 14 was 52 years old at the time of the study. Interestingly, in 2 subsequent studies, T → A transversions, such as seen in patient 14, accounted for only 3 out of 103 of all age-related *TET2* mutations [36, 37], and so it seems that this patient’s mutation is distinct from the phenomenon of age-related clonal hematopoiesis. It seems more likely that that this patient’s *TET2* mutation provides an advantage for the stem cell clone in a manner that does not completely compete against the normal stem cell pool and which is dependent upon an abnormal stem cell environment—as is thought to be the case for the PNH clone [14, 38].

It is notable that practically 100% of the red cells and granulocytes in patient 14 are GPI-negative, whereas the *TET2* mutant allele burden is < 50% in granulocytes and nucleated red cells, approximately 50% in monocytes, and undetectable amongst GPI (+) and GPI (−) lymphocytes. These findings suggest that the *TET2*-mutated population belongs to a subclone. Notably, the *TET2* mutant clone and the PNH population are both large and stable in relative size in this patient, in contrast to *TET2* mutations reported previously in patients with bone marrow failure [39–41]. The simplest explanation for the findings is that first, a PNH stem cell population expanded, followed by expansion of a *TET2* mutant clone, which arose out of a subset of the PNH stem cells. It is possible that the *TET2* mutation is not evident among lymphocytes because it is instead driving the stem cell towards monocytic differentiation. The fact that the *TET2* mutation was not seen in either the GPI (+) or GPI (−) lymphocyte population strongly suggests that the mutation is not in the germline.

An alternative model, proposing that hypermutability could contribute to the generation of somatic mutations in *PIG*-*A* in PNH [42, 43] could explain why some patients have several distinct PNH clones [44–46], and in some cases, mutations in genes other than *PIG*-*A*. Indeed, in PNH and aplastic anemia, in addition to *JAK2*, *TET2* and *HMGA2* (mentioned above), mutations in *ASXL1*, *DNMT3a*, *BCOR*, *BCORL1*, *SUZ12*, and *U2AF1* have been reported [39–41]. However, considering that *TET2* can be inactivated by a single mutation, and given that haploinsufficiency for *TET2* can drive clonal expansion [31], and given that *TET2* and *PIG*-*A* have a comparable number of codons, we believe that if hypermutability were fundamental to PNH, then *TET2* would have been commonly mutated in our cohort of patients as well. Our data, then, is more consistent with experimental models suggesting that the mutation rate is normal in PNH [47–49], and we believe that the association with aplastic anemia and the immune escape model explains clonal expansion in most patients. However, recent reports of PNH arising in the setting of a CALR-mutated MPN [50],CML [51] and a relapse of AML associated with *TET2* and *JAK2* mutations [52] raise the question of whether autoimmunity is necessary in all cases. The elucidation of the autoantigen in aplastic anemia and the identification of the responsible T cell clones may shed light on this question in exceptional cases such as these and the one described here [53].

## Conclusions

Based on a large cohort of patients with highly expanded PNH clones, we conclude that mutations in *TET2* can contribute to clonal expansion in exceptional cases. Our findings argue against the hypermutability model in most patients with PNH.

## Data Availability

The datasets used and/or analused during the current study are available from the corresponding author on reasonable request

## Abbreviations

PNH: paroxysmal nocturnal hemoglobinuria
GPI: glycosylphosphatidylinositol
MPN: myeloproliferative neoplasm
AA: aplastic anemia
